# Medical Examination of Schools and Scholars

**Published:** 1911-06

**Authors:** 


**Medical Examination of Schools and Scholars.** Edited by T. N. KELYNACK, M.D. Pp. xvi., 434. London: P. S. King & Son. 1910.

As is pointed out by Sir Lauder Brunton in the Introduction, "this volume consists of a collection of studies by experts," and in consequence it has many of the advantages and some of the defects which one would expect to find in such a collection.

At a time when the results of medical inspection of school children in England are to a large extent a problem of the future, the various authors have had to confine their remarks in the main either to an exposition of the various parts of the Education (Administrative Provisions) Act of 1907, or to an account of the methods in which they have themselves set about carrying out the provisions of that Act. There are, in addition, many interesting chapters dealing with voluntary medical inspection in preparatory and secondary schools, and the progress of medical inspection in various foreign countries, notably the United States of America and Germany.

It is unfortunate that the degree of detail with which each subject is treated varies considerably. The very important question of the correlation of the School Medical Service with the Public Health Service is dealt with in eight pages, though it might have been expanded with very great advantage, *e.g.* an attempt might have been made to define the responsibilities of each service in the matter of infectious diseases in schools, a point which is not at all clear in the various circulars and memoranda which have been issued from the Medical Department of the Board of Education. The chapter dealing with the very important subject of "Dental Conditions in Elementary School Children" seems also far too brief for a volume of this size. On the other hand, the article on "Eyes and Eyesight of School Children" forms a treatise of forty pages of very valuable information, which cannot possibly be utilised to its fullest advantage by the ordinary medical inspector (as distinct from the expert ophthalmic inspector), who has to examine from twenty to thirty children in one school attendance.

It is interesting to note in the chapter on "The General

Routine Medical Examination of School Children" that the writer insists that medical inspectors should be men or women of wide clinical experience—surely a plea that they should be selected from those who are actually engaged in consulting or general practice.

Amongst others, those chapters dealing with "Medical Inspection in Secondary Schools," "Medical Supervision of Games, etc.," "Mentally Defective Children," "School Clinics," "The Feeding of School Children," and "Open-air Schools," form very interesting reading.

